# The influence of vision on tactile Hebbian learning

**DOI:** 10.1038/s41598-017-09181-6

**Published:** 2017-08-22

**Authors:** Esther Kuehn, Juliane Doehler, Burkhard Pleger

**Affiliations:** 1 Department of Neurology, Max Planck Institute for Human Cognitive and Brain Sciences, Leipzig, 04103 Germany; 2 Center for Behavioral and Brain Sciences Magdeburg, Magdeburg, 39106 Germany; 3 Aging and Cognition Research Group, DZNE, Magdeburg, 39106 Germany; 4 Institute of Psychology, Leipzig University, 04109 Leipzig, Germany; 5 Department of Neurology, BG University Hospital Bergmannsheil, Ruhr-University Bochum, Bochum, 44789 Germany

## Abstract

NMDA-dependent Hebbian learning drives neuronal plasticity in different cortical areas, and across species. In the primary somatosensory cortex (S-I), Hebbian learning is induced via the persistent low-rate afferent stimulation of a small area of skin. In particular, plasticity is induced in superficial cortical layers II/III of the S-I cortex that represents the stimulated area of skin. Here, we used the model system of NMDA-dependent Hebbian learning to investigate the influence of non-afferent (visual) input on Hebbian plasticity in S-I. We induced Hebbian learning in 48 participants by applying 3 hours of tactile coactivation to the right index fingertip via small loudspeaker membranes. During coactivation, different groups viewed either touches to individual fingers, which is known to activate S-I receptive fields, touches to an object, which should not activate S-I receptive fields, or no touch at all. Our results show that coactivation significantly lowers tactile spatial discrimination thresholds at the stimulated finger post- versus pre-training across groups. However, we did not find evidence for a significant modulatory effect of visual condition on tactile spatial discrimination performance. This suggests that non-afferent (visual) signals do not interact with Hebbian learning in superficial cortical layers of S-I, but may integrate into deeper cortical layers instead.

## Introduction

In the primary somatosensory cortex (S-I), NMDA (N-methyl-D-aspartate)-dependent Hebbian learning is induced by constant low-rate stimulation from the thalamus. If this input is combined with a depolarization of the postsynaptic cell, long-term-potentiation (LTP) is induced^1, 2^. The activity by a nearby location induces a postsynaptic depolarization, the direct low-rate input then leads to LTP^3^.

In rodents, S-I firing rates of non-trimmed whiskers already increase after 20 minutes of coactivation, more after 2 hours, and significantly after 6 hours^4^. In addition, learning effects are larger when whiskers are stimulated passively than when they are used actively, which is likely due to the effect of correlated activity^4^, and points to implicit learning. NMDA-dependent receptor density is least in cortical layer IV and greatest in the most superficial neocortical layers^5^. In addition, adult S-I plasticity mostly occurs in cortical layers II and III, but not in layer IV^6^. NMDA-induced S-I plasticity therefore seems to be confined to a small, localizable area, that is, to layers II and III of the S-I cortex that represents the coactivated area of skin. This renders coactivation-induced S-I plasticity an ideal model system not only in animals but also in humans to study precise signal integration in S-I during tactile learning.

Prior studies in humans showed that observing touches to individual fingers activates the corresponding S-I receptive field of the actual finger^7^. For example, observing an index finger being touched by a brush activates the same S-I receptive field as real brush strokes applied to the same finger^7^. Conversely, observation of object touch is not related to enhanced S-I activity^8^. However, it is an unanswered question at which cortical layer visual signals are integrated in S-I. Both deep and superficial cortical layers of S-I offer input structures that in principle allow the integration of visual signals^9^. Because deep and superficial cortical layers have different functional roles^9–13^, answering the ‘cortical depth question’ of visual-to-tactile integration is an important prerequisite for understanding the functional role of visual signals in S-I, and the influence of multisensory input on cortical layer-dependent sensory plasticity in general. Here, we argue that investigating the specific influence of visual signals on NMDA-dependent Hebbian learning that in animals is confined to superficial cortical layers in S-I provides a valuable first step to target these questions.

To this end, we induced Hebbian learning in S-I using an established tactile coactivation protocol^14^. This protocol applies weak tactile stimuli to a small area of skin for three hours. This leads to significantly improved tactile spatial discrimination thresholds for the coactivated area as tested with the two-point discrimination task (2PDT)^15–18^. Prior studies showed that after coactivation, tactile spatial discrimination thresholds improve at the stimulated finger, but not at the neighboring fingers, or the same finger of the other hand^15^. Similar to rodents, coactivation therefore induces plasticity in humans only at the receptive field that is stimulated. In addition, coactivation does not lead to improved tactile spatial discrimination thresholds when NMDA-receptors are blocked using a NMDA-receptor blocker, but effects are doubled by amphetamine, as one would expect for NMDA-dependent learning^14^. This provides additional evidence for a similar role of NMDA in human S-I Hebbian learning as has been reported in rodents.

To study the effect of vision on tactile Hebbian learning in S-I, we applied coactivation to participants’ right index fingertips for three hours while they underwent different visual stimulus conditions. During coactivation, one group observed index finger touches on a screen (“social group”), a second group observed object touches on a screen (“object group”), and a third group just looked at a fixation cross (“no-vision group”). Before and after coactivation, participants were tested in their individual two-point discrimination thresholds using a fully automated testing device. We expected a main effect of training due to the known beneficial effect of tactile coactivation on spatial tactile discrimination thresholds^14, 15, 19, 20^. In addition, if visual signals integrated into superficial cortical layers II/III of S-I receptive fields, we expected an interaction between visual and tactile signals, and a significantly higher learning effect in the social group compared to both the object group and the no-vision group. Conversely, if visual signals integrated into deeper cortical layers V/VI of S-I receptive fields, no such modulatory effect of vision on tactile learning was expected. Finally, if there was an effect of visual congruency on tactile learning, that is, a beneficial effect of observing synchronous object movements for tactile learning (note that the social group and the object group observed touches that were applied congruently to the physical touches they perceived), we expected the social and the object group to show significantly higher learning effects than the no-vision group. The results of our study offer valuable initial information on the influence of non-afferent (visual) signals on layer-dependent plasticity in S-I.

## Results

### Effect of vision on tactile learning

We tested N = 48 participants (age range = 18–30 years, 24.81 ± 2.84 years (mean ± SD), 24 females), all of them were healthy, none had any history of neurological or sensory impairments, and all had normal or corrected-to-normal vision. According to the Edinburgh questionnaire^21^, all subjects were right-handed.

**Figure 1:**
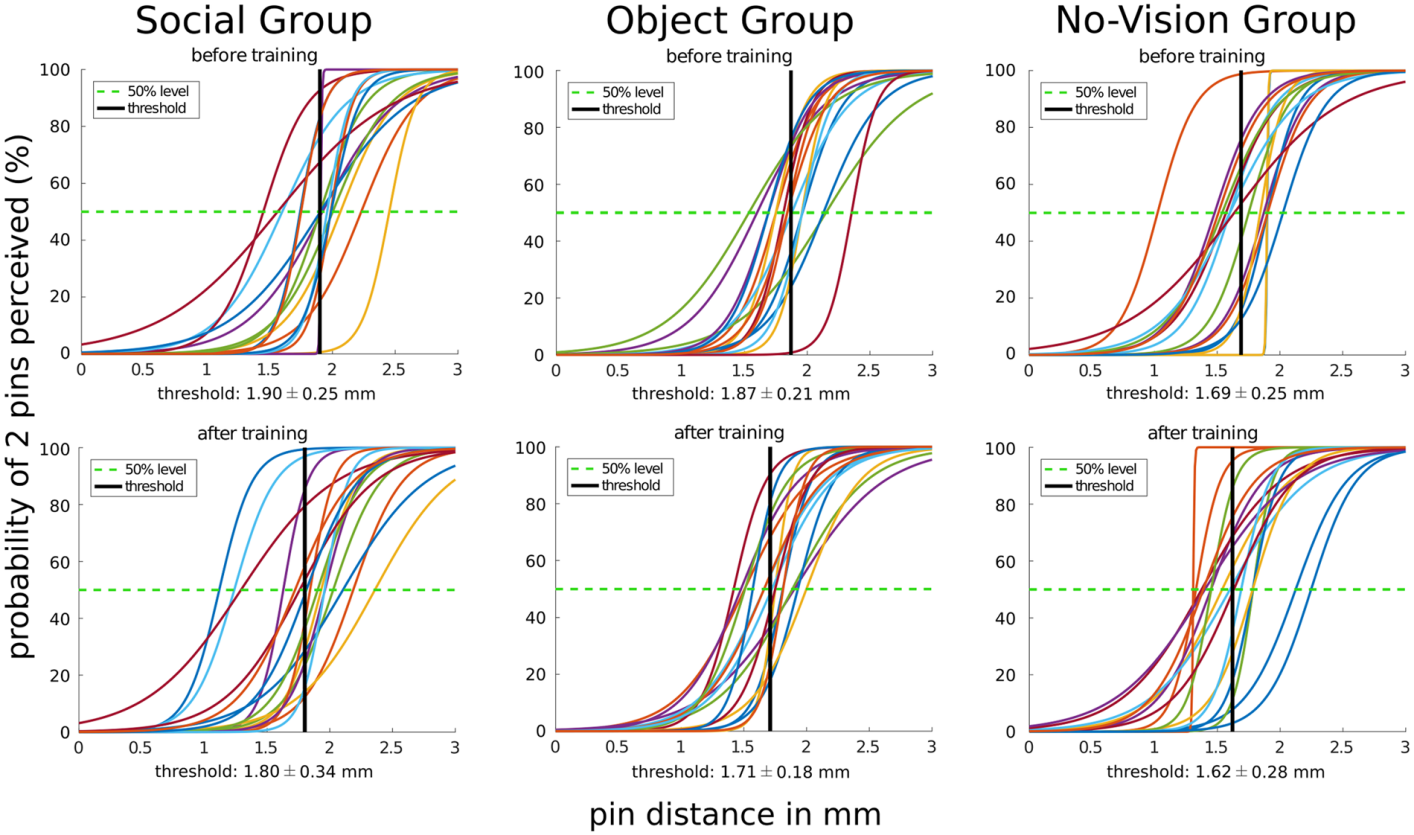
Fitted psychometric functions of N = 48 individual participants who took part in the initial training experiment (n = 16 in each group). Probabilities of “two” - responses are plotted against ascending pin distances. Group mean two-point discrimination thresholds at the 50% probability level are indicated as black lines. Sigmoid curves are plotted with a sampling rate of 0.01 mm within a range from 0 to 3 mm.

**Figure 2:**
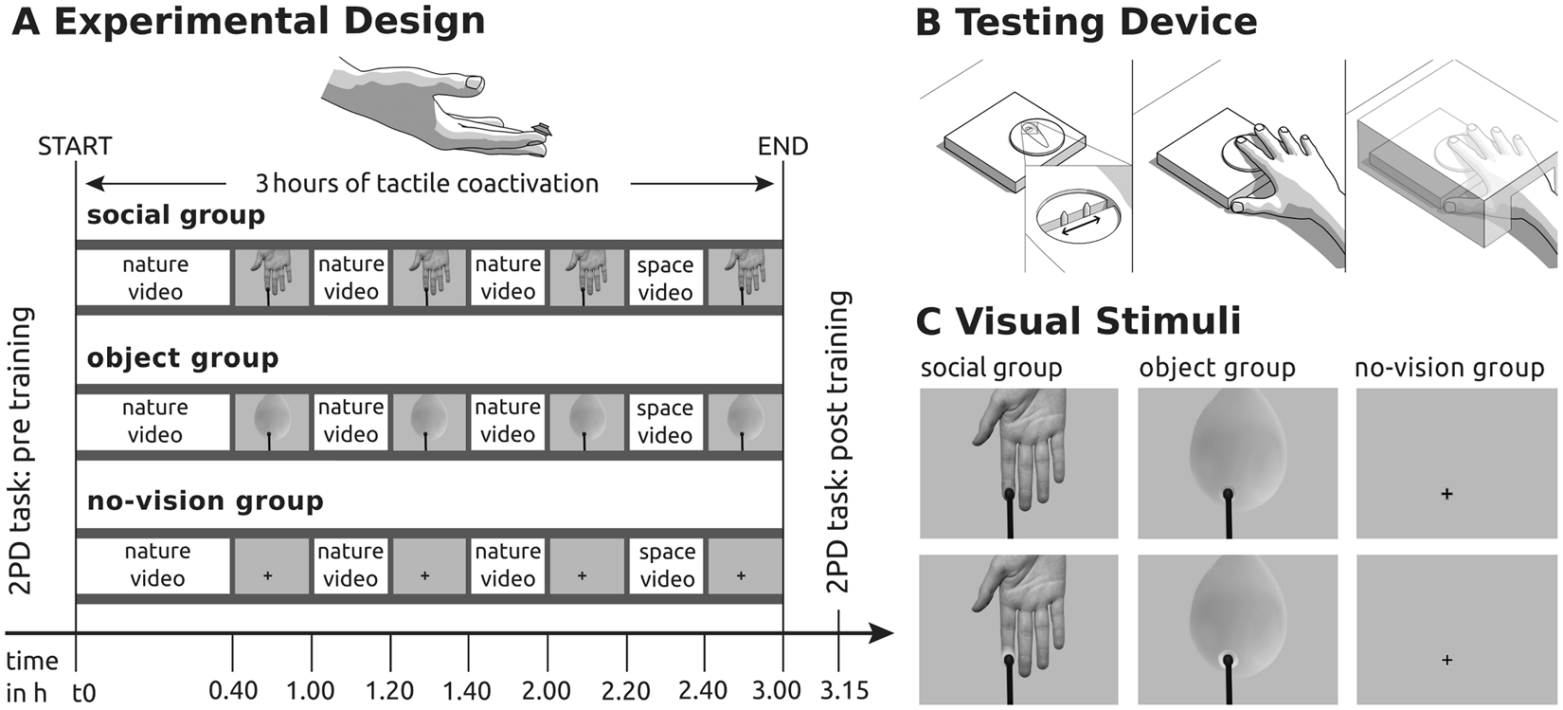
Stimuli and experimental design. (**A**) The two-point discrimination task (2PD task) was applied once before and once after training (pre, post). Training comprised three hours of tactile coactivation applied by a small loudspeaker membrane attached to the right index fingertip (see schematic drawing upper panel). During tactile coactivation, all participants watched nature and space videos, or were exposed to different visual stimulus conditions (four blocks, 20 minutes each, also see (**C**)). Participants of the “social group” watched synchronous touches to a hand, participants of the “object group” watched synchronous touches to a balloon, participants of the “no-vision group” saw a fixation-cross that changed in colour. Time is given in hours (h). (**B**) Testing device. A fully automated Piezo-electric stimulator was used to measure two-point discrimination thresholds. During the 2PD task, vision of the right hand was precluded by a white box. (**C**) Enlarged presentation of the visual stimulus material as introduced in (**A**).

**Figure 3:**
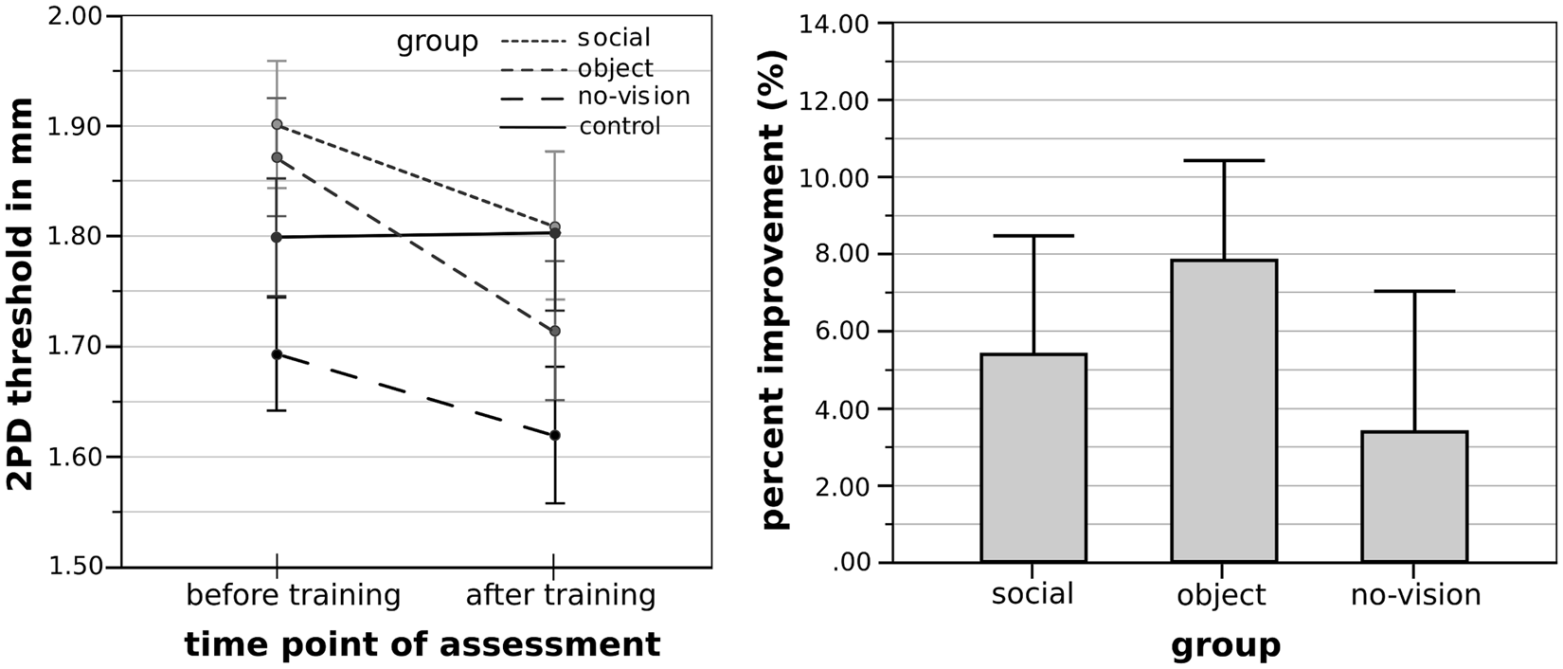
Effect of three hours of tactile coactivation on two-point discrimination thresholds. Left panel: Two-point discrimination thresholds of the three different groups of the initial training experiment (social, object, no-vision), and of the control group (control experiment 1) that did not receive training. Note that lower thresholds indicate better performance. Right panel: Bar plots show training effects (post – pre, in %) in two-point discrimination thresholds for the three groups of the initial training experiment. Shown are means and standard errors (SEs).

We conducted an ANOVA taking individual two-point discrimination thresholds at the 50% level (see individual plots in Fig. 1) as dependent variable with the factors group (social, object, no-vision), and training (pre, post). We found a main effect of training (*F*(1,45) = 12.28, *p* = 0.001, *ƞ*² = 0.21) and a trend towards a main effect of group (*F*(2,45) = 3.07, *p* = 0.056, *ƞ*² = 0.12; social group: 1.85 ± 0.28 mm; object group: 1.79 ± 0.17 mm; no-vision group: 1.65 ± 0.23 mm), but no interaction between group and training (*F*(2,45) = 0.63, *p* = 0.54, *ƞ*² = 0.03). The main effect of training (pre vs post) was due to a significantly lower two-point discrimination threshold at the tip of the right index finger after the tip of the right index finger had been coactivated for three hours compared to before the training across groups (two-point discrimination threshold pre = 1.82 ± 0.25 mm, post = 1.71 ± 0.28 mm, difference pre – post = 0.11 ± 0.22 mm; see Fig. 2 for design and stimuli). We also investigated the effect of group on training by conducting a one-way ANOVA with the factor group (social, object, no-vision) with training effect (in %) as dependent variable. There was no main effect of group (*F*(2,45) = 0.51, *p* = 0.60, see Fig. 3).

We also conducted ANOVAs taking the two-point discrimination thresholds at the 25% and 75% levels (rather than the 50% level, see above) as well as response variability (steepness of the curve = difference 75% threshold − 25% threshold) as outcome measures with the factors group (social, object, no-vision) and training (pre, post). We found a main effect of training for the two-point discrimination thresholds at the 25% and 75% levels (25%: *F*(1,45) = 11.28, *p* = 0.002, *ƞ*² = 0.20; 75%: *F*(1,45) = 7.17, *p* = 0.010, *ƞ*² = 0.14), a main effect of group taking the 75% threshold but not the 25% threshold as dependent variable (25%: *F*(2,45) = 2.09, *p* = 0.136, *ƞ*² = 0.09; 75%: *F*(2,45) = 3.53, *p* = 0.038, *ƞ*² = 0.14), and, importantly, again no interaction between group and training using both measures (25%: *F*(2,45) = 0.43, *p* = 0.654, *ƞ*² = 0.02; 75%: *F*(2,45) = 0.52, *p* = 0.600, *ƞ*² = 0.02). The main effect of training (pre vs post) was due to a significantly lower two-point discrimination threshold at the tip of the index finger after the tip of the index finger had been coactivated for three hours compared to before the training across groups (25%: two-point discrimination threshold pre = 1.62 ± 0.29 mm, post = 1.50 ± 0.29 mm, difference pre – post = 0.12 ± 0.25 mm; 75%: two-point discrimination threshold pre = 2.02 ± 0.25 mm, post = 1.92 ± 0.30 mm, difference pre – post = 0.10 ± 0.25 mm). The main effect of group at the 75% threshold level was due to a significantly lower two-point discrimination threshold in the no-vision group compared to the social group (social group = 2.07 ± 0.29 mm, object group = 1.99 ± 0.18 mm, no-vision group = 1.85 ± 0.22 mm; social vs object: *t*(30) = 0.84, *p* = 0.406; social vs no-vision: *t*(30) = 2.38, *p* = 0.024; object vs no-vision: *t*(30) = 2.01, *p* = 0.054). There was no effect of training, no effect of group, and no interaction between group and training for response variability (all p > 0.45).

### Attention to visual stimuli during training

During training, participants were asked to count the number of stronger touches (for social group and object group, see Fig. 2C lower versus upper panels) or the number of times the fixation cross changed colour (for no-vision group). Performance in this task provided us with an individual measure of attention to the visual stimuli during training. All three groups showed high performance in this task (mean % correct responses across groups: 90.12%, *Mdn* = 91.13%). An independent-sample Kruskal Wallis H-test for non-normal data with the factor group (social, object, no-vision) and the dependent variable detection ability (in %) was not significant (*χ*²(2) = 4.52, *p* = 0.105; social group: mean rank score = 18.88, *Mdn* = 87.63%; object group: mean rank score = 25.38, *Mdn* = 91.75%; no-vision group: mean rank score = 29.25, *Mdn* = 92.50%).

### Control experiment 1: No significant training effect without coactivation

Because we did not find a modulatory effect of group (i.e., visual condition) on tactile learning (i.e., two-point discrimination thresholds post- versus pre-training), one possible interpretation of our results is that participants improved just as an effect of time. To exclude this possibility, we conducted a second experiment where we invited 16 novel participants (24.38 ± 2.92 years, 8 females) and tested their two-point discrimination thresholds using the same procedure as described above for the no-vision group, but without applying tactile coactivation to the index fingertip. Here, we did not find improvements in two-point discrimination thresholds after three hours, in fact, thresholds were numerically worse (pre = 1.80 ± 0.18 mm, post = 1.81 ± 0.24 mm, difference pre – post = −0.01 ± 0.17 mm). We conducted a paired sample t-test and compared thresholds pre versus post and did not obtain a significant difference (*t*(15) = −0.22, *p* = 0.83).

### Control experiment 2: Increasing the sample size increases the p-value for interaction between group and training

Because we did not find a modulatory effect of group on tactile learning in our first experiment, another possible interpretation of our results is that the sample size (n = 16 per group) was too small to observe a significant influence of vision on tactile learning. Even though our sample size was motivated by a prior study^22^, we invited 32 novel participants (24.38 ± 2.92 years, 16 females) who took part in a third experiment either as social group (n = 16), or as object group (n = 16) to explore this possibility. We conducted two-by-two ANOVAs taking individual two-point discrimination thresholds at the 50% level as dependent variable with the factors group (social, object) and training (pre, post) both for the original sample size (N = 32) and for the new, doubled sample size (N = 64).

As expected, we found a main effect of training for both sample sizes (n = 32: *F*(1,30) = 12.494, *p* = 0.001, *η*² = 0.294; n = 64: *F*(1,62) = 16.532, *p* = 0.00014, *η*² = 0.211), which was due to a significantly lower two-point discrimination threshold at the right index fingertip after the tip of the right index finger had been coactivated for three hours compared to before the training across groups (n = 32: two-point discrimination threshold pre = 1.89 ± 0.23 mm, post = 1.76 ± 0.27 mm, difference pre – post = 0.13 ± 0.20 mm; n = 64: two-point discrimination threshold pre = 1.84 ± 0.24 mm, post = 1.74 ± 0.24 mm, difference pre – post = 0.10 ± 0.20 mm). For the main effect of training, increasing the sample size therefore *de*creased the p-value. However, we found neither a main effect of group (n = 32: *F*(1,30) = 0.579, *p* = 0.453, *η*² = 0.019; n = 64: *F*(1,62) = 0.004, *p* =0 .949, *η*² ≤ 0.001) nor an interaction between group and training (n = 32: *F*(1,30) = 0.656, *p* = 0.424, *η*² = 0.021; n = 64: *F*(1,62) = 0.104, *p* = 0.748, *η*² = 0.002) for both sample sizes. For the main effect of group, and for the interaction between group and training, increasing the sample size *in*creased the p-value.

### Bayesian statistics

As conventional significance testing is not sensitive to gain support for the null hypothesis of no effect, we also conducted Bayesian statistics on the larger data set as acquired during control experiment 2 (see above). Bayesian statistics provide degrees of beliefs (Bayesian probabilities) to explain a state of the world, rather than detailing whether or not two data sets differ significantly. Because we did not find significant differences between the object group and the social group in learning, we additionally calculated the Bayesian probability (Bayes factor) as relative measure of similarity. For comparison, we also calculated the Bayes factor for the main effect of training across groups.

**Figure 4:**
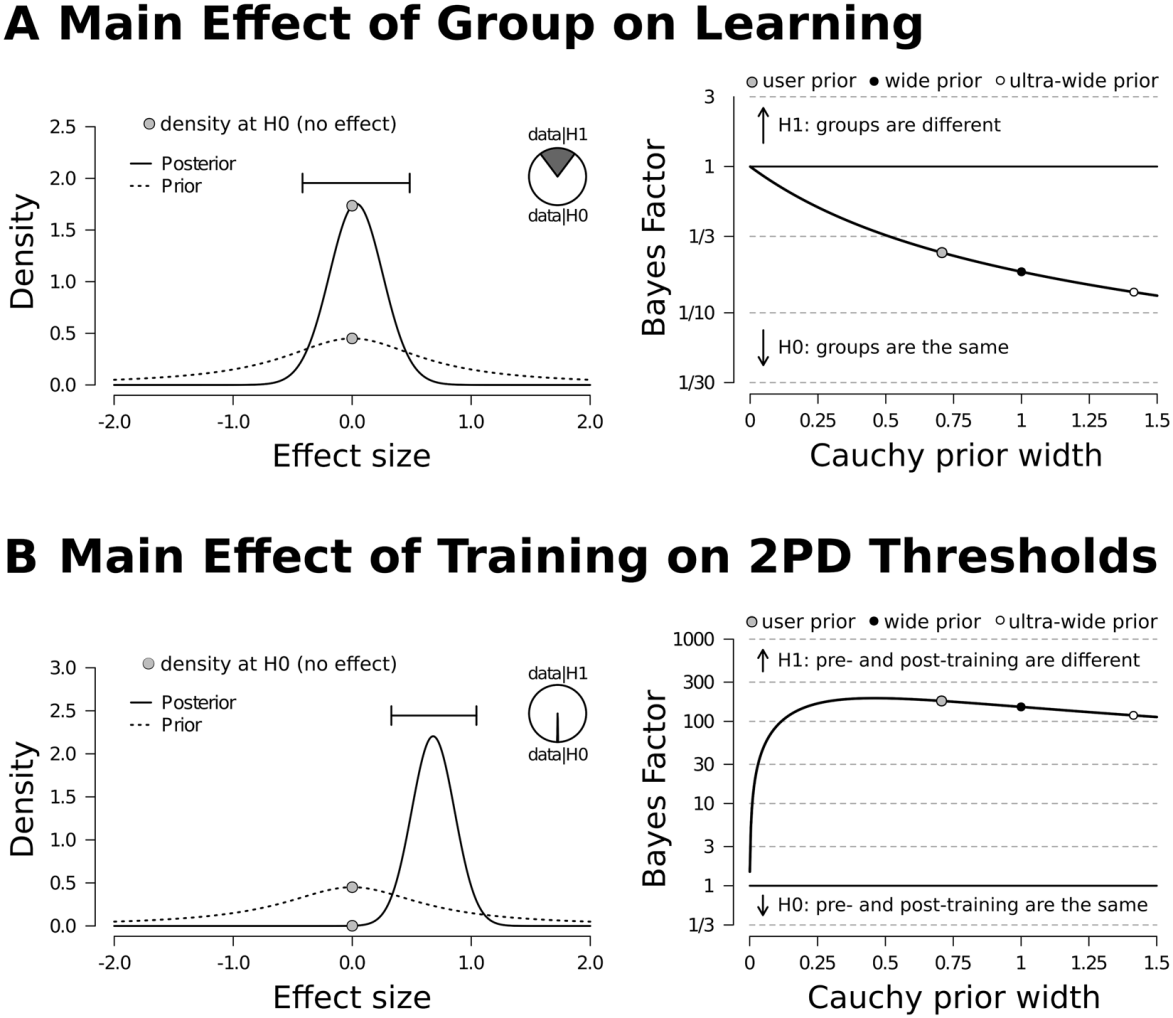
Overview of Bayesian statistics. The prior distributions are shown as dashed lines, and the posterior distributions are shown as solid lines. The grey dots indicate the height of the curves (density) at the null hypothesis (H0). The robustness plots (right side) for the chosen priors (default value = 0.707) show how the Bayes factor (y-axis) changes for a wide range of prior width (x-axis). A Bayes factor of 1 or greater represents evidence in favor of the alternative hypothesis (H1), and a Bayes factor below 1 represents evidence in favor of the H0. (**A**) A Bayesian independent samples t-test was calculated to test the main effect of group on learning. The higher dot on the posterior distribution indicates that the Bayes factor supports the H0 (groups are the same). When testing the robustness of the results, the width of the prior increases, whereas the Bayes factor decreases, indicating that also wider priors represent evidence in favor of the H0. (**B**) A Bayesian paired samples t-test was calculated to test the main effect of training. The higher dot on the prior distribution indicates that the Bayes factor supports the H1 (two-point discrimination thresholds differ between pre- and post-training measurement). In this case, when testing robustness of the result, the width of the prior increases and the Bayes factor decreases, which supports the H1 as it always stays above 100.

With respect to the first analysis, the alternative hypothesis (significant differences between groups in learning) was predicted 0.26 times better than the null hypothesis (no differences between groups in learning) when testing the difference of learning effects between groups (see Fig. 4A). The error associated with the Bayes factor was 0.002%. We observed a learning effect of 5.25 ± 11.19% for the object group and a learning effect of 4.83 ± 11.13% for the social group. With respect to the second analysis, the alternative hypothesis (significant differences as a main effect of training) was predicted 177.10 times better than the null hypothesis (no main effect of training) when testing the effect of tactile coactivation on two-point discrimination thresholds (see Fig. 4B). The error associated with the Bayes factor was less than 0.001% (4.45 * 10^−9^).

### Questionnaire items

To investigate the subjective experience of training, after the experiment we also asked participants to fill out a questionnaire that assessed different aspects of body ownership, body perception, and touch perception. Questionnaire items and answers to questionnaire items are summarised in Table 1. As most of the items were non-normally distributed, we used non-parametric test procedures to compare values statistically. Importantly, participants felt as if the observed touches occurred at the same time as the physical touches (social group: *Mdn* = 2, *Z* = 2.56, *p* = 0.010; object group: *Mdn* = 3, *Z* = 2.99, *p* = 0.003, values compared against zero), whereas the colour change of the cross (no-vision group) did not induce the feeling of visuo-tactile congruency (*Mdn* = −2, *Z* = −3.45, *p* = 0.001, values compared against zero), as was intended by our experimental design.

**Table 1:** Summary of questionnaire items and answers.

Questionnaire items	Group Values (Median) One-sample tests (against zero)			Group Comparisons Independent-sample tests		
	social group (n = 16)	object group (n = 16)	no-vis. group (n = 16)	social vs object group	social vs no-vis. group	object vs no-vis. group
1: “It looked as if the observed hand/balloon was touched”.	**Mdn** = **2**	**Mdn** = **2**	N/A	Z = −0.77	N/A	N/A
	Z = 1.34	Z = 1.74		p = 0.468		
	p = 0.180	p = 0.082				
2: “It looked as if the stick was stopped in front of the observed hand/balloon”.	**Mdn** = **−2**	**Mdn** = **−2**	N/A	Z = −1.08	N/A	N/A
	Z = −3.28	Z = −1.81		p = 0.305		
	p = 0.001	p = 0.070				
3: “It felt as if the observed touch/colour change occurred at the same time as the touch I felt (meaning both touch events occurred at the same time)”.	**Mdn** = **2**	**Mdn** = **3**	**Mdn** = **−2**	Z = −0.06	Z = −4.06	Z = −4.47
	Z = 2.56	Z = 2.99	Z = −3.45	p = 0.956	p = 0.00002	p = 6.5e-7
	p = 0.010	p = 0.003	p = 0.001			
4: “The observed hand/balloon began to resemble my own hand”.	**Mdn** = **−1**	**Mdn** = **−3**	N/A	Z = 3.63	N/A	N/A
	Z = −1.86	Z = −3.61		p = 0.0003		
	p = 0.063	p = 0.0003				
5: “It felt as if the observed hand/balloon were my hand”.	**Mdn** = **−2**	**Mdn** = **−3**	N/A	Z = 2.15	N/A	N/A
	Z = −2.80	Z = −3.67		p = 0.043		
	p = 0.005	p = 0.0002				
6: “It seemed as if the observed touch caused the touch I felt”.	**Mdn** = **−1**	**Mdn** = **−1**	N/A	Z = −0.25	N/A	N/A
	Z = −2.65	Z = −1.56		p = 0.809		
	p = 0.008	p = 0.119				
7: “For me the observed hand/balloon looked artificial”.	**Mdn** = **−2**	**Mdn** = **3**	N/A	Z = −3.89	N/A	N/A
	Z = −1.02	Z = 3.49		p = 0.00006		
	p = 0.307	p = 0.0005				
8: “For me the observed hand/balloon looked realistic”.	**Mdn** = **2**	**Mdn** = **−2**	N/A	Z = 4.11	N/A	N/A
	Z = 2.39	Z = −3.39		p = 0.000009		
	p = 0.017	p = 0.001				
9: “When I observed the hand/balloon/fixation cross, it felt as if my own hand was numb”.	**Mdn** = **−1**	**Mdn** = **−2**	**Mdn** = **0**	Z = 0.25	Z = 0.43	Z = 0.56
	Z = −1.92	Z = −2.19	Z = −1.47	p = 0.809	p = 0.696	p = 0.590
	p = 0.055	p = 0.028	p = 0.142			
10: “When I observed the hand/balloon/fixation cross, I felt a tingling sensation in my right index finger”.	**Mdn** = **−1**	**Mdn** = **−2**	**Mdn** = **0**	Z = 1.61	Z = −0.52	Z = 1.11
	Z = −1.34	Z = −2.96	Z = −1.64	p = 0.119	p = 0.616	p = 0.287
	p = 0.180	p = 0.003	p = 0.100			
11: “The touch to my finger felt the same as it looked like in the video”.	**Mdn** = **−1**	**Mdn** = **0**	N/A	Z = −0.73	N/A	N/A
	Z = −1.53	Z = −0.57		p = 0.491		
	p = 0.125	p = 0.571				
12: “When I watched the videos of the hand/balloon/fixation cross, I did not know exactly where my own hand was”.	**Mdn** = **−2**	**Mdn** = **−2**	**Mdn** = **−2**	Z = 0.54	Z = −0.20	Z = 0.16
	Z = −3.44	Z = −3.30	Z = −2.80	p = 0.616	p = 0.867	p = 0.897
	p = 0.001	p = 0.001	p = 0.005			

Participants were asked to rate 12 (social group and object group), or 4 (no-vision group) statements on a bipolar seven-point scale ranging from −3 (strong contradiction) to +3 (strong agreement). Group values are given as group medians (Mdn). We calculated non-parametric one-sample Wilcoxon signed rank-tests to test group medians against zero (neutral answer), and independent-sample Mann-Whitney U-Tests to compare item distributions between groups. Standardised test statistics (Z) and p-values (p) are given for computed tests.

## Discussion

Here, we target the ‘cortical depth question’ of visual-to-tactile integration by using the model system of coactivation-induced Hebbian learning in S-I. We induced Hebbian learning in three groups of participants by applying weak tactile stimuli to a confined area at the tip of the index finger. During tactile coactivation, one group observed touches applied to the tip of the index finger (“social group”), one group observed touches applied to an object (“object group”), and a third group simply looked at a fixation cross (“no-vision group”). As expected, we found a main effect of training across groups, as evidenced by improved tactile spatial discrimination thresholds for the stimulated finger post-training versus pre-training. However, we did not observe an interaction between group and training, and did not find a main effect of group on the training effect. This pattern appeared using different performance thresholds of the psychometric function (25%-, 50%-, and 75%-thresholds). In animals, NMDA-dependent Hebbian learning in S-I is confined to superficial cortical layers of the S-I cortex that represents the coactivated body part^5, 6^. If this pattern transfers to humans, our data suggest that visual signals do not (or only weakly) interact with tactile signals in superficial cortical layers during Hebbian learning, but may be integrated into deeper cortical layers instead. This is in accordance with previous studies on the modulatory influence of vision on tactile perception (outlined below). Our results provide initial insights into the influence of multisensory integration on cortical depth-dependent sensory plasticity in humans.

NMDA-receptor density is greatest in the most superficial neocortical layers, and least in the input layer IV^5^. The fast S-I responses to touch (5–8 ms) are therefore not NMDA-mediated, whereas the later responses (20–100 ms) are^23^. In adults, S-I plasticity mostly occurs in cortical layers II and III, but not in the input layer IV^6^. This suggests a specific modulation of superficial cortical layers of the S-I cortex that represents the stimulated area of skin during tactile coactivation. When finger touch is observed, single finger receptive fields in S-I are activated^7^. It is not clear to date, however, at which cortical layer visual signals arrive in S-I, and whether they interfere with local neuronal interactions that occur at superficial cortical layers during learning. If tactile input is combined with a depolarization of the postsynaptic cell induced by vision, LTP should be induced^1, 2^. Our data do not provide empirical evidence that visual signals ameliorate tactile Hebbian learning in S-I, which suggests either an integration of visual signals into deeper cortical layers in S-I, or an interaction that is too weak to be detected with our measures.

An integration of visual signals into deeper cortical layers in S-I would be in accordance with studies that showed a modulatory influence of vision on tactile spatial discrimination thresholds^24, 25^ – a process assumed to be mediated by intracortical inhibition^26, 27^. Similar modulatory influences have been assigned to deep cortical layers^28^. In animals, attentional modulation is known to primarily affect the firing rates of neurons in superficial cortical layers of S-I, and a positive effect of attentional modulation on NDMA-dependent tactile plasticity has been assumed^29^. And indeed, whereas we did not find an influence of vision on tactile Hebbian learning in S-I, modulatory influences of attention on two-point discrimination thresholds exist^30^. If these initial assumptions were to be confirmed by future research, this would indicate a possible dissociation of modulatory influences on signal processing in S-I: whereas attentional modulation would primarily affect tactile processing in superficial cortical layers, non-afferent (visual) signals would primarily affect tactile processing in deep cortical layers. Neuroimaging studies using high field strength (i.e., 7 Tesla and above) in combination with behavioral assessments^7, 31–33^ will have to be used to test these specific hypotheses.

Additionally, it is important to note that absence of evidence should not be equated with evidence of absence. It remains possible that whereas in the here presented paradigm no significant effect of vision on tactile Hebbian learning could be detected, this is due to the specific experimental design and/or analyses methods chosen rather than due to the absence of any influence of vision on S-I-mediated tactile Hebbian learning. Other paradigms may vary the length of the visual stimulation, the pattern of tactile stimuli applied during vision, and/or the visual stimuli themselves to investigate the robustness of our finding across experimental designs. In addition, even though learning effects were not significantly different between groups, there may be an effect of vision on somatosensory neuronal firing rates that did not transfer to the behavioral outcome measure, i.e., the two-point discrimination threshold. The conclusions drawn from these data therefore remain preliminary.

Taken together, we here offer a first approach to the question of how visual signals interact with local tactile Hebbian learning. Our behavioral data provide first evidence towards signal integration into deeper cortical layers due to a lack of modulatory influence on tactile Hebbian learning that is assumed to take place in superficial cortical layers. However, further studies using neuroimaging methods such as layer-dependent structural mapping^34^ and layer-dependent fMRI^31, 32^ will have to investigate this question further. Our paradigm may offer a valuable behavioral marker for neuronal plasticity characterizations.

## Materials and Methods

### Participants

n = 48 participants took part in the initial training experiment (age range = 18–30 years, 24.81 ± 2.84 years (mean ± SD), 24 females). Additionally, n = 16 novel participants took part in control experiment 1 conducted to show that tactile coactivation is mandatory to induce tactile learning (age range = 20–29 years, 24.38 ± 2.92 years, 8 females). Additionally, n = 32 novel participants took part in control experiment 2 conducted to replicate parts of our effects with larger sample sizes (age range = 19–29 years, 24.50 ± 2.50 years, 16 females). In summary, N = 96 participants (age range = 20–29 years, 24.38 ± 2.92 years, 48 females) were tested in our three experiments. All of them were healthy, none had any history of neurological or sensory impairments, and all had normal or corrected-to-normal vision. According to the Edinburgh questionnaire, all subjects were right-handed^21^. Professional musicians were a priori excluded due to the influence of musical training on sensorimotor plasticity^22, 35, 36^. All participants were paid for their attendance (7.00 EUR per hour; except for n = 25 participants who received 9 EUR per hour after an institute-wide increase of remuneration). Participants gave informed consent prior to each experiment. The Ethics committee of the Leipzig University approved the study, and all methods were performed in accordance with the ethical guidelines and regulations as offered by the Leipzig University, and the Max Planck Institute for Human Cognitive and Brain Sciences, Leipzig.

### Assessment of two-point discrimination thresholds

The two-point-discrimination task (2PDT) was used to estimate individual two-point discrimination thresholds at the right index fingertip. We used a fully automated, custom-built testing device for this purpose (QuaeroSys Piezo Stimulator, see Fig. 2). The stimulator is driven by a piezo-motor, which automatically raises two pins at a fixed height (1 mm) and at a fixed speed (1000 ms). This device is improved compared to most other 2PDT testing devices, because pins are not applied manually to the skin, but are applied automatically (i.e., with controlled speed, pressure, and height). During testing, the two pins were applied to the index fingertip at distances ranging from 0.7 to 2.5 mm, in increments of 0.3 mm. No one-pin condition was included to avoid participants simply using the metric of salience as answer criterion. In neurophysiological studies, it was shown that two points evoke a different number of action potentials in cutaneous mechanoreceptive afferents of rhesus monkeys than one point^37^. Stimulation was provided from below while participants’ right hands rested comfortably on the testing device, the index fingertip above a small whole (see Fig. 2). Stimulation was applied to participants’ index fingertips of the right hand, just below the finger pad. Each distance was presented 10 times in a pseudorandomized order with inter-trial intervals (ITIs) between 1000 and 5000 ms (randomized and counter-balanced across distances), adding up to 70 trials in total. After each pin application, participants had to decide if they felt the sensation of one or two pins (two-alternative forced-choice task). Participants were instructed to only respond “two” if they clearly felt two separate pins touching their skin. Responses were given by pressing a foot pedal. Half of the participants used their left foot to respond “one” and their right foot to respond “two”, the other half used the right foot to respond “one” and the left foot to respond “two”. To prevent effects caused by seeing the stimulated finger on tactile discrimination thresholds^25, 38^, the right hand was covered by a white box during the experiment. Participants were provided with earplugs to avoid influences of sound on task performance.

### Procedure

Participants were first invited to an initial assessment. Here, they were first familiarised with the experiment, and were then asked to discriminate the two most extreme distances from each other (i.e., 0.7 mm from 2.5 mm). If they succeeded in this task, the full 2PDT was run three times in a row. This took about 8 minutes per block, approximately 30 minutes in total. We only invited participants to the training experiment whose two-point discrimination thresholds of the three runs in the test session varied less (i.e., showed a lower standard deviation) than the expected effect size of tactile coactivation on two-point discrimination thresholds (i.e., 0.2 mm, see refs 14–19, 39). In addition, we used goodness-of-fit estimates to exclude participants who did not show the expected logistic regression response (see below for detailed explanation of the statistical analyses). Out of N = 101 participants that we invited to an initial assessment, n = 48 participants took part in the training experiment (47.52%).

### Tactile co-activation training

The training took place on a separate day (usually less than 1 week after initial assessment). Here, the 2PDT was applied once before and once after training (i.e., tactile coactivation). We ensured via multiple visual markers on participants’ fingers that the same area of skin was tested before and after training. In addition, the markers ensured that it was always the tested area that was stimulated via coactivation. Tactile coactivation was applied via a small loudspeaker membrane (diameter = 14.8 mm, see Fig. 2). The loudspeaker membrane was mounted on the tip of the right index finger to convey weak tactile stimuli to a small area of skin for three hours (see Fig. 2). The loudspeaker was computer-controlled via the software tool Presentation (version 16.5, Neurobehavioral Systems, Inc., Albany, CA, USA). Co-activation-pulses were presented at different ITIs between 100 to 3000 ms in a pseudorandomised order. The average stimulation frequency was 1 Hz, and the duration of each pulse was 10 ms. The tactile coactivation protocol followed a previously validated protocol^14–17, 19, 20, 22^. Tactile stimuli were applied at supra-threshold intensities. During the tactile stimulation period, participants underwent different visual stimulus conditions (see below).

### Visual stimulus conditions

Participants were divided into three groups of 16 participants each (each group half male, half female). All groups underwent three hours of tactile coactivation as described above. During coactivation, four 20-minute blocks of visual stimulation were presented (see Fig. 2). During these blocks, the social group observed touches to another person’s (gender-matched) hand, the object group observed touches to an (size- and colour-matched) object, and the no-vision group observed a fixation cross on the screen positioned at a location corresponding to the location where the observed touches were presented. In humans, visually perceived touch peaks in S-I with a latency of 150 ms after visual stimulation onset^40^. Activation of S-I responses driven by touch occurs at about 50 ms from tactile stimulus onset^41, 42^. To ensure that visual and tactile signals arrived congruently in S-I, the difference between both processing durations (i.e., 100 ms) was used to calculate stimulus onset asynchrony, that is, the visual signals were presented 100 ms earlier than the tactile signals.

Each 20-minute block was divided into 18 one-minute stimulation phases. In each stimulation phase, some of the visual touches were stronger (i.e., applied with more force) than others (see Fig. 2). After each stimulation phase, participants were asked how often the hand/object received stronger touches (possible numbers: 2/3/4 times, randomised and counterbalanced across conditions; see Fig. 2C). Participants of the no-vision group counted the number of colour changes of the fixation-cross instead (also 2/3/4 times, randomised and counterbalanced across conditions; see Fig. 2). Answers were given via a foot pedal. Feedback (in % correct performance) was provided to all participants after each block. Colour changes were not synchronised to tactile events and were randomly presented within each visual stimulation block. All participants wore earplugs during the visual blocks. Tactile coactivation was applied for three hours, whereas the visual blocks covered four times 20 minutes (80 minutes in total). In the remaining time period (100 minutes), all participants watched the same nature^43–45^ and space^46^ videos to provide the same visual input to all participants during this time. The videos showed natural scenes, for example mountains, clouds, forests, or lakes, as well as views of the earth from space, but did not show any mammals, or humans (neither auditorily nor visually), or any type of social interaction. All videos showed rather neutral content (i.e., they were not expected to trigger strong emotional responses).

### Post-inquiry questionnaire

Immediately after the experiment, participants filled out a paper-pencil questionnaire. Item construction and combination was based on previous literature^47, 48^. Items are listed in Table 1.

### Control experiment 1 without coactivation

To ensure that the observed learning effect was driven by plasticity as induced by tactile coactivation and that it was not a simple effect of time, we conducted a second experiment where we invited 16 novel participants to undergo a similar protocol to the no-vision group, but they were not coactivated. We expected no learning in this group.

### Control experiment 2 with increased sample size

To investigate the influence of sample size on the observed effects, we invited 32 novel participants to take part. They underwent the assessment and the training either as social group (n = 16) or as object group (n = 16). We conducted ANOVAs taking the two-point discrimination threshold at the 50% level as independent variable with the factors group (social, object) and training (pre, post) both for the original and the doubled sample size.

### Statistical analyses

All responses were plotted against pin distances, and psychometric functions were generated via the Statistics and Machine Learning toolbox as implemented in Matlab (version 9.1, The MathWorks Inc., Natick, MA, 2016). Data were fitted via the glmfit function, which offers an iterative weighted least square (IWLS) algorithm to receive maximum-likelihood estimators. IWLS is a standard procedure to fit generalised linear models^49^. Here, we used a binary logistic regression to fit the data, that is, “two”-responses were fitted as percentages across ascending pin distances (0.7–2.5 mm). The individual two-point discrimination thresholds were taken from the pin distance where the 25%, 50% and 75% levels crossed the fitted sigmoid curve. We evaluated goodness-of-fit for the logistic regression model using deviance test statistics, i.e., the likelihood ratio test. In generalised linear models, deviance computation is a common test statistic to compare a given model, for example the fitted model with the saturated model^50–52^. Large values provide evidence for a lack of adequacy between data and model (for detailed description and formula see^50, 52^). Here, we compared the deviance of the fitted model with the deviance of the null model (the null model is the most restricted model that contains no additional predictors), which is a common statistical procedure to test goodness-of-fit for logistic regression models^50, 51, 53^. Under the null hypothesis, the difference between the deviance of the null model and the deviance of the fitted model is chi-square distributed^53^. To test whether the null hypothesis has to be rejected, we calculated the p-value for the deviance difference. A low p-value indicates a high probability that an observed deviation from the null model is significant. A significance level of 15% was determined as cut-off criterion^54^. Only participants whose response data led to an adequate model fit indicated by a p-value lower than 0.15 were reinvited to the main test. Such less conservative alpha error probabilities between 10 and 20% are commonly used in goodness-of-fit tests^55^.

The discrimination thresholds at 25%, 50%, and 75% probability levels as well as the response variability (difference between the 75% and 25% thresholds) were used as dependent variables in a mixed-effect ANOVA that we conducted with the factors group (social, object, no-vision) and training (pre, post). In addition, we estimated individual training success by subtracting pre-training thresholds from post-training thresholds. These values were normalised (in %) and used as independent variables in a one-way ANOVA with the factor group (social, object, no-vision). We used a significance level of p < 0.05 to estimate significant main effects, and interactions. Analysed samples were tested for normality using Shapiro Wilk’s test. In case of skewed ordinal data, corresponding non-parametric test procedures were used. As the ANOVA is to some extent robust against violations of normality^56, 57^, we used ANOVAs because only a minority of the two-point discrimination data was skewed (object group: 75% threshold pre-training: *W*(16) = 0.83, *p* = 0.008, response variability pre-training: *W*(16) = 0.87, *p* = 0.028; no-vision group: 75% threshold pre-training: *W*(16) = 0.87, *p* = 0.032, 50%-threshold pre-training: *W*(16) = 0.89, *p* = 0.047).

The JASP software package (version 0.8.1.2; JASP Team, 2017) was used for the Bayesian analysis of the data. We calculated a Bayesian independent samples t-test on the learning effect with group (social, object) as between-subjects factor, and a Bayes factor favoring the alternative hypothesis. Additionally, we calculated a Bayesian paired samples t-test over all subjects (N = 64) with time of measurement (pre, post) as within-subjects factor to provide a reference to the reader on how the Bayes factor relates to the p-values of the respective analysis. Again, we used a Bayes factor favoring the alternative hypothesis. For both analyses, we did not model the expected effect size, but selected the default Cauchy-scaled prior of 0.707. However, we simulated the effect of the prior on the Bayes factor for a wider range of prior width to estimate how robust the conclusions were to the chosen prior.

### Data availability

The datasets generated during and/or analysed during the current study are available from the corresponding author on reasonable request.
